# Coarctation of the Aorta: An Atypical Case Treated by a Double Layer Stent Technique

**DOI:** 10.1016/j.ejvsvf.2022.02.005

**Published:** 2022-02-23

**Authors:** Jose Damian Herrera Mingorance, Ana María Pérez Bailón, Jose Maria Moreno Escobar, Luis Miguel Salmerón Febres

**Affiliations:** a Hospital Universitario Clínico San Cecilio, Granada, Spain; b Hospital Universitario Virgen de las Nieves, Granada, Spain

**Keywords:** Coarctation of the aorta, Coral reef aorta, Endovascular repair

## Abstract

**Introduction:**

Coarctation of the aorta (CoA) is one of the more common congenital heart defects that usually manifests in adults as poorly controlled hypertension. When technically possible, the treatment of choice for adult CoA is an endovascular approach with covered stent placement. A case is presented with atypical clinical onset, treated endovascularly with a double layer stent technique.

**Report:**

A 41 year old previously asymptomatic woman with an unremarkable past medical history presented with sudden dyspnoea, unstable blood pressure and pulse, and a radial femoral systolic pressure difference of 53 mmHg. A computed tomography scan showed coral reef aorta: aortic stenosis from a highly calcified lesion located distal to the origin of the left subclavian artery, compatible with CoA. Within a few hours, the patient went rapidly into cardiogenic shock with multiple organ failure requiring urgent intervention. Using a dual left iliac conduit and right brachial artery access, the lesion was pre-dilated with an 8 × 60 mm balloon. A double layer technique was then applied by coaxially deploying a BeGraft aortic stent (expanded to 18 mm) followed by a Conformable GORE® TAG® thoracic stent graft (26 × 26 × 100 mm). The patient's symptoms improved and the radial femoral systolic gradient decreased to 12 mmHg.

**Discussion:**

Sudden onset CoA is a rare condition in adults that can lead to refractory cardiogenic shock and multiple organ failure. In anatomically complex cases, a double layer technique may be beneficial because it has high radial force and good wall apposition with lower risk of stent collapse than single stent deployment.

## Highlights

• Coarctation of the aorta can have a sudden presentation and lead to cardiogenic shock.
• The pathology can be caused by a very calcified aortic lesion (coral reef aorta).
• Endovascular treatment is the preferred approach in symptomatic adults.
• A dual layer technique may have a lower risk of stent collapse.

## Introduction

Coarctation of the aorta (CoA) is a common condition in infants with heart disease, with an estimated prevalence of 5%–8%.^1^ In adults, CoA may be due to a native lesion or restenosis of a repaired lesion. Coarctation consists of narrowing of the aorta, typically at the area of insertion of the ductus arteriosus and very rarely ectopically (in the ascending, descending, or abdominal aorta).

In adults, CoA is often an asymptomatic, incidental finding, although it occasionally presents as refractory hypertension. Concomitant comorbidities include bicuspid aortic valve in more than half of patients,^2^ and intracranial and aortic aneurysms. Aortic dissection may be associated with CoA in the setting of poorly controlled hypertension. Hypertension, which often persists after successful aortic repair, can in turn cause myocardial infarction, cerebrovascular disease, and heart failure.^3^

Surgical coarctation repair was first performed in 1945; these patients’ prognosis has improved significantly since then.^4^ Progress in endovascular techniques has made covered stent deployment the current treatment of choice in adults, when technically possible^5^ because of its improved short and medium term outcome.^6^ The objective of this report is to describe this novel double layer technique in a highly calcified aortic lesion distal to the left subclavian artery.

## Case report

A 41 year old woman with a history of recurrent migraine, with no other illnesses or previous symptoms, was admitted urgently to the Intensive Care Unit with sudden and intense dyspnoea, with significant respiratory distress and without associated chest pain. On hospital arrival, she was unstable with a supraventricular tachycardia (170 bpm), a blood pressure of 156/95 mmHg in the right arm, 88% saturation with an oxygen reservoir bag, peripheral hypoperfusion in the lower limbs with bilateral absent femoral pulses, and paraesthesia with difficulty mobilising and weakness in both lower limbs. Upper limb perfusion was normal and radial and ulnar pulses were present and symmetrical.

**Figure 1:**
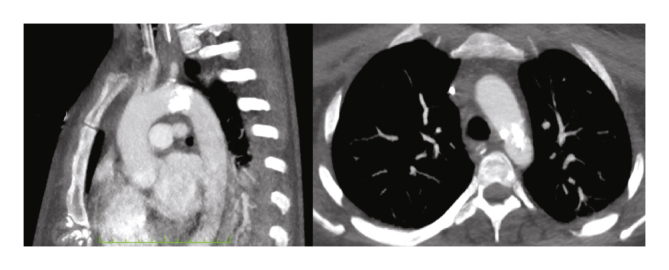
Coral reef aorta immediately distal to the origin of the left subclavian artery.

The patient was sedated, intubated, and started on mechanical ventilation. A computed tomography scan of the chest, abdomen, and pelvis revealed coral reef aorta immediately distal to the origin of the left subclavian artery, with a large coral shaped plaque causing critical stenosis in that area (Fig. 1). Occlusion of the superior mesenteric artery origin was also noted. In addition, evidence suggesting heart failure was found, with hepatic congestion, low contrast uptake in both kidneys, lower limbs, and intestinal wall, bilateral pleural effusion, and several areas of atelectasis.

A transoesophageal echocardiogram confirmed the presence of the coral reef aortic lesion, which was causing a severe pressure gradient and a low ejection fraction (30%). The radial femoral systolic pressure gradient was 53 mmHg (150–97 mmHg).

During diagnostic work up, the patient went into cardiogenic shock, requiring high dose inotropic agents, with multiple organ failure and associated paraparesis. An urgent intervention was performed in the operating theatre using a retroperitoneal approach and a 10 mm diameter Dacron conduit to gain access to the left iliac artery, due to the small calibre of the femoral accesses. A retrograde puncture was made in the iliac conduit and a short 7Fr sheath was inserted. Access was then gained via the right brachial artery with a short 5Fr sheath and a guidewire was advanced to the abdominal aorta. The guidewire was snared and exteriorised through the introducer inserted in the iliac conduit to achieve a through and through manoeuvre and establish an “arterial railway”.

**Figure 2:**
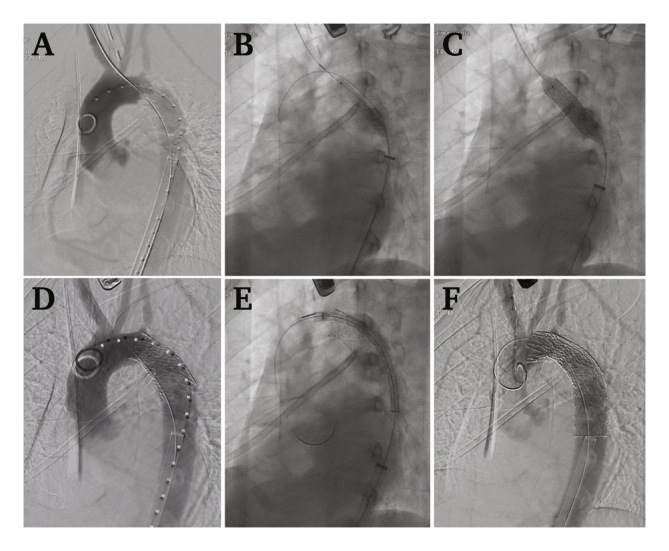
A: Initial arteriogram showing coral reef aortic lesion causing severe stenosis. Through and through manoeuvre from right brachial access and left iliac conduit. B: Pre-dilatation of the lesion with an 8 × 60 mm balloon. C: BeGraft covered stent deployment, expanded to 18 mm. D: Angiogram after BeGraft deployment showing bird's beak appearance. E: Thoracic aortic stent graft progression over a Lunderquist guidewire in the ascending aorta. F: Final angiogram after deployment of the aortic stent graft.

The iliac conduit sheath was exchanged for a 20 F Gore DrySeal sheath and an angiogram was performed with a pigtail catheter from the ascending aorta, visualising the origin of the supra-aortic vessels and the coral reef aortic plaque and associated stenosis (Fig. 2A). First, the aortic lesion was dilated with an 8 × 60 mm angioplasty balloon (Fig. 2B) and then a covered BeGraft aortic stent (18 × 48 mm) was inserted and dilated to 18 mm in the affected area (Fig. 2C), achieving good expansion and no signs of contrast extravasation on repeat angiogram, but with poor apposition to the inner arch curvature (bird's beak effect) (Fig. 2D).

Then, using a double layer technique, a Conformable GORE® TAG® thoracic stent graph (26 × 26 × 100 mm) was deployed inside the covered stent, distal to the left carotid artery, with intentional covering of the left subclavian artery. The repeat angiogram showed no technical defects. The stent graft was released over a 0.035 Lunderquist guidewire with the end in the ascending aorta (losing the through and through manoeuvre), to allow conformability of the stent (Fig. 2E and F) and dilated close to its nominal size in the lesion area. Finally, from the brachial access, the mesenteric artery lesion was recanalised, deploying a 7 × 56 mm balloon expandable stent.

**Figure 3:**
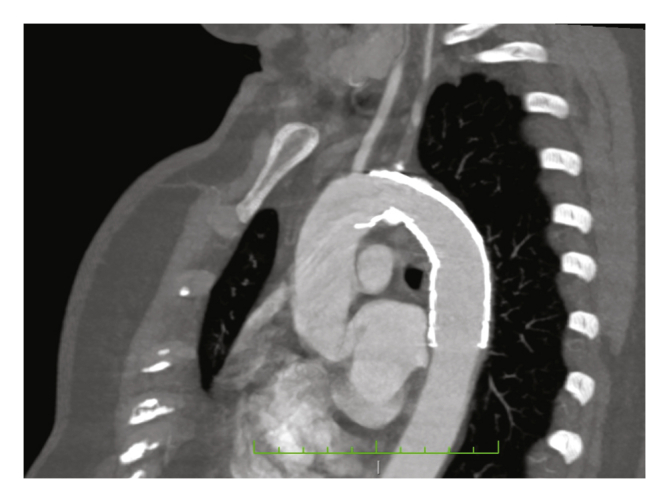
Follow up CT scan (2 months).

The patient made a good cardiological and haemodynamic recovery, allowing vasoactive drugs to be discontinued 24 hours post-operatively. The systolic pressure gradient was 12 mmHg. She contracted nosocomial pneumonia, which responded to antibiotic therapy. The patient remained paraparetic after surgery, and returned to her referral hospital for paraparesis rehabilitation, with good recovery after two months (able to walk with a walker, almost total recovery of sensitivity with feet paraesthesia). A computed tomography scan was performed two months after surgery showing no device complications (Fig. 3), and at the eight month visit the patient remained asymptomatic and no re-interventions have been required.

## Discussion

This case report has two main points of interest. The first is the atypical presentation of this condition in the patient (sudden onset of multiple organ dysfunction syndrome despite having a highly calcified lesion, from which a chronic course would be expected), and the second is the double layer aortic coverage not previously described in the literature.

In adults, CoA (as a *de novo* finding or as re-coarctation) refers to evidence in imaging studies of aortic narrowing associated with refractory systemic hypertension, lower limb claudication, and ischaemic nephropathy.

The European Society of Cardiology Guidelines for the treatment of adult congenital heart disease recommend repair of coarctation in hypertensive patients with an increased non-invasive gradient between upper and lower limbs confirmed with invasive measurement (peak to peak >20 mmHg), with catheter treatment (stenting) the preferred technique when technically feasible.^5^ According to the results of the COAST II (Coarctation of the Aorta Stent Trial) study, covered stents are preferable to bare metal stents because they have fewer complications in the short and medium term.^6^^,^^7^ Balloon angioplasty should be reserved for dilatation of previously stented lesions and for patients unsuitable for stent placement and surgery.^8^

It was decided to first deploy a balloon expandable covered aortic stent and then an aortic stent graft. The aim of the short balloon expandable covered stent was to provide the precision and radial force needed to dilate the heavily calcified lesion while protecting against the risk of an aortic tear. The purpose of the stent graft, with its enhanced conformability, was to better adapt to the lesser curvature of the aortic arch, improving proximal fixation. Risk of aortic graft (self expandable) collapse is a recognised complication (especially in cases of excessive oversizing, acute arch angulation, or traumatic injuries), but the collapse of balloon expandable stents is also a possible risk. Early and late cases of stent collapse are described in the literature,^9^ and in those cases malapposition to the aortic wall was a common factor, leading to a high transmural gradient across the stent wall (greater than the radial strength of the stent) resulting in infolding and stent collapse.^10^ Therefore, apposing the upstream stent edge to the aorta and avoiding residual gradients is extremely important.

Using this double layer technique was planned from the beginning. Due to the lesion location, the bird's beak effect was very likely to occur if a single stent technique was used. Treating very complex or calcified lesions – such as the patient's coral reef aorta – with this double layer technique, has not been reported previously in the literature.

Finally, the patient's paraparesis was attributed to her haemodynamic shock and multiple organ failure (kidney and liver failure, and lower limb ischaemia), which she had on admission, rather than as a complication of the intervention. The suspicion of the haemodynamic cause of the paraparesis was corroborated by the lower limb hypomobility in particular – already present before sedating the patient – together with the short length of the covered aortic stent, the patent and lesion free supra-aortic vessels, and the rarity of paraparesis in CoA. In this case, the left subclavian artery was not revascularised because of the short aortic coverage (and low probability of paraplegia), the focal aortic lesion, the patency of supra-aortic trunks and circle of Willis and the haemodynamic instability during the procedure.

This double layer technique may be beneficial in those cases where high radial force and good conformability are required, as in highly calcified aortic lesions distal to left subclavian artery.

## Conclusion

In adults, CoA can have sudden onset of symptoms and lead to cardiogenic shock and multiple organ failure in previously asymptomatic patients. When treating complex aortic lesions, the dual layer technique may have the benefit of a better aortic wall apposition and a lower risk of stent collapse, as well as a lower risk of re-coarctation compared with single stent deployment.

## References

[1] Tynan M., Finley J.P., Fontes V., Hess J., Kan J. (1990). Balloon angioplasty for the treatment of native coarctation: results of valvuloplasty and angioplasty of congenital anomalies registry. Am J Cardiol.

[2] Aboulhosn J., Child J.S. (2006). Left ventricular outflow obstruction: subaortic stenosis, bicuspid aortic valve, supravalvar aortic stenosis, and coarctation of the aorta. Circulation.

[3] Roos-Hesselink J.W., Schölzel B.E., Heijdra R.J., Spitaels S.E.C., Meijboom F.J., Boersma E. (2003). Aortic valve and aortic arch pathology after coarctation repair. Heart.

[4] Crafoord C., Nylin G. (1945). Congenital coarctation of the aorta and its surgical treatment. J Thorac Surg.

[5] Baumgartner H., De Backer J., Babu-Narayan S.V., Budts W., Chessa M., Diller G.-P. (2021). 2020 ESC Guidelines for the management of adult congenital heart disease. Eur Heart J.

[6] Taggart N.W., Minahan M., Cabalka A.K., Cetta F., Usmani K., Ringel R.E. (2016). Immediate outcomes of covered stent placement for treatment or prevention of aortic wall injury associated with coarctation of the aorta (COAST II). JACC Cardiovasc Interv.

[7] Ringel R.E., Vincent J., Jenkins K.J., Gauvreau K., Moses H., Lofgren K. (2013). Acute outcome of stent therapy for coarctation of the aorta: results of the coarctation of the aorta stent trial. Catheter Cardiovasc Interv.

[8] Stout K.K., Daniels C.J., Aboulhosn J.A., Bozkurt B., Broberg C.S., Colman J.M. (2019). 2018 AHA/ACC guideline for the management of adults with congenital heart disease: a report of the American College of Cardiology/American Heart Association Task Force on Clinical Practice Guidelines. Circulation.

[9] Saran M., Sasidharan B., Sivasubramonian S. (2017). Late stent collapse in aortic coarctation—an interplay of residual pressure gradient and radial strength. IHJ Cardiovasc Case Rep.

[10] Hayes N., Podnar T., Qureshi S. (2014). Collapse of the advanta V12 large diameter covered stent following implantation for aortic coarctation. Catheter Cardiovasc Interv.

